# Ectopic expression of a *Brassica rapa AINTEGUMENTA* gene (*BrANT-1*) increases organ size and stomatal density in Arabidopsis

**DOI:** 10.1038/s41598-018-28606-4

**Published:** 2018-07-12

**Authors:** Qian Ding, Bing Cui, Jingjuan Li, Huayin Li, Yihui Zhang, Xiaohui Lv, Nianwei Qiu, Lifeng Liu, Fengde Wang, Jianwei Gao

**Affiliations:** 1 Institute of Vegetables and Flowers, Shandong Academy of Agricultural Sciences and Shandong Key Laboratory of Greenhouse Vegetable Biology and Shandong Branch of National Vegetable Improvement Center, Jinan, 250100 China; 2 College of Life Sciences, Shandong Normal University, Jinan, 250014 China; 3 College of Life Sciences, Qufu Normal University, Qufu, 273165 China

## Abstract

The AINTEGUMENTA-like (AIL) family plays a central role in regulating the growth and development of organs in many plants. However, little is known about the characteristics and functions of the AIL family in Chinese cabbage (*Brassica rapa* L. ssp. *pekinensis*). In this study, a genome-wide analysis was performed to identify the members of the AIL family in Chinese cabbage. We identified three *ANT* genes and six *ANT-like* genes of Chinese cabbage, most of which were differentially expressed in different organs or tissues. Furthermore, compared with the wild-type line, the size of different organs in the *35S-BrANT-1* line was significantly increased by promoting cell proliferation. Meanwhile, over-expression of *BrANT-1* also increases the stomatal number and delays the leaf senescence. Transcriptome analyses revealed that a set of cell proliferation and stoma development genes were up-regulated, while the senescence-associated genes were down-regulated, suggesting these genes may be involved in *BrANT-1* regulated processes for controlling organ size, stomatal density and leaf senescence. In summary, this study offers important insights into the characteristics and functions of the *ANT* genes in Chinese cabbage, and provides a promising strategy to improve yield or head size in Chinese cabbage breeding programs.

## Introduction

The size of the leafy head is an important economical trait of Chinese cabbage (*Brassica rapa* L. ssp. *pekinensis*). However, little is known about how the size of the leafy head is controlled. Therefore, understanding the molecular mechanism of the leafy head development has always been a key goal of genetic improvement in Chinese cabbage.

Plant organ size reflects both cell number and cell size which are regulated by a polygenic system through cell proliferation and cell expansion^1,2^. Previous reports have shown that many regulatory factors are involved in these two processes^3^, such as *AINTEGUMENTA* (*ANT*)^4–7^, *ANT-like* (*AIL*)^8,9^, auxin-regulated gene involved in organ size (*ARGOS*)^10,11^ and growth-regulating factors (*GRFs*)^12,13^. ANT-like (AIL) transcription factors are members of the APETALA2 (AP2) subfamily, belonging to the APETALA2/ETHYLENE RESPONSE FACTOR (AP2/ERF) super family^14^. The AP2 subfamily is divided into two groups, euAP2 group and ANT group. the euAP2 group is characterized by a microRNA binding site (*miR172*) in the post-domain region, and the ANT group is characterized by lineage-specific motifs or amino acid insertions in the two AP2 domains. The ANT group is further subdivided into basal ANT lineage and euANT lineage, according to the length of the pre-domain region sequences and other specific motifs. For example, the euANT lineage has a long pre-domain region (127–307 aa) and three specific motifs in the pre-domain region (the euANT2, 3, and 4 motifs: WLGFSLS, PKLEDFLG, and TFGQR), while the basal ANT lineage has a short pre-domain region (44–81 aa)^15^. A wealth of studies have shown that the AIL gene family members are involved in the ovule primordium initiation, female gametophyte formation, as well as organ growth and polarity^4,5,7,16^. For example, the transgenic plants over-expressing the *ANT* genes result in the production of organs with a large size^7,17,18^, while the loss-of-function mutations of the *ANT* gene have a smaller organ size^4,5,7^. Additionally, many studies revealed that *ANT* regulates organ size by changing the total cell numbers^7^. Furthermore, cells ectopically expressing *ANT* in fully differentiated organs exhibit neoplastic activity by producing calli and adventitious roots and shoots^7^. These studies strongly suggest that *ANT* most likely maintains ongoing cell proliferation coordinately with cell growth^19^. However, to our knowledge, the AIL family from Chinese cabbage has not been characterized. Therefore, identifying and analyzing the AIL family in Chinese cabbage is of great interest.

In this study, we investigated the BrAIL family members in Chinese cabbage through genome-wide bioinformatics analysis, including the identification and characterization of the AIL family members, gene structural analysis, phylogeny and motif analysis. The expression patterns of these genes were characterized in detail in response to the naphthaleneacetic acid (NAA) treatment in different tissues by quantitative real-time PCR (qRT-PCR). Our results show *BrANT-1* had the highest sequence similarity to *AtANT* (AT4G37750), and hence was chosen for further functional analysis. Over-expression of *BrANT-1* enhanced organ size by increasing cell number and stomatal density and delaying leaf senescence. Furthermore, by analyzing the potential pathways where *BrANT-1* participates, it is possible to understand how the gene regulates Chinese cabbage yield and/or head size.

## Results

### Identification of AIL family members in Chinese cabbage

A total of nine BrAIL family members were identified in the Chinese cabbage genome, according to the taxonomy of the AP2 superfamily (Supplementary Tables S1 and S2). These included three *ANT* genes and six *AIL* genes (Table 1). The sequences of each member were downloaded from the Brassica database (http://brassicadb.org/brad/)^20^ and the two AP2 domains were confirmed according to the SMART database (http://smart.embl-heidelberg.de/). All nine BrAIL family members were named by the *A*. *thaliana* orthologs based on the sequence similarity of the protein sequences. The detailed description of sequence similarity between *Arabidopsis thaliana* and *Brassica rapa* is shown in Supplementary Table S3. When more than two Chinese cabbage genes were mapped to one homologous gene in *A*. *thaliana*, one additional number was added to the end of the gene name^21^. For example, three *ANT* genes, Bra017852, Bra011782 and Bra010610, were homologs of *AtANT* (AT4G37750). Accordingly, they were named *BrANT-1*, *BrANT-2* and *BrANT-3*, respectively. The BrAIL family members were randomly mapped to different chromosomes of *B*. *rapa* (chromosome number: 01, 02, 03, 06, 08 and 10). The chromosome 02 and 03 contained three and two *BrAIL* genes, respectively, while the chromosome 01, 06, 08 and 10 contained only one *BrAIL* gene. Subsequent sequence analysis showed that the coding sequences of these nine *BrAIL* genes had a length of 1323 to 1725 bp, which encode a peptide of 440 to 574 aa. The encoded proteins had a predicted molecular weight varying from 49.6 to 64.1 kDa and a theoretical isoelectric point (pI) varying from 6.09 to 7.81.

**Table 1:** Summary of Chinese cabbage AIL genes and proteins.

Transcription factor name (*B*.*rapa*)	Gene name	Chromosome(Strand)	Gene start/stop	CDS(bp)	length(aa)	MW(KDa)	pI
BrANT-1	Bra017852	A03(+)	31016132/31018580	1671	556	61.3	6.81
BrANT-2	Bra011782	A01(−)	568506/570905	1638	545	60.4	7.81
BrANT-3	Bra010610	A08(+)	14745020/14747414	1662	553	61.3	6.64
BrAIL1	Bra008040	A02(+)	11786723/11790469	1323	440	49.6	6.26
BrAIL5	Bra020444	A02(+)	5780684/5783620	1680	559	60.8	7
BrAIL6-1	Bra028584	A02(+)	1611866/1615493	1692	563	63.3	6.4
BrAIL6-2	Bra006065	A03(+)	1837427/1841214	1671	556	62.5	6.09
BrAIL6-3	Bra009026	A10(−)	13718237/13723014	1725	574	64.1	6.24
BrAIL7	Bra024394	A06(+)	16095323/16098499	1350	449	50.5	6.32

### Phylogenetic relationships and gene structure of the AIL protein in *Arabidopsis*, rice and Chinese cabbage

**Figure 1:**
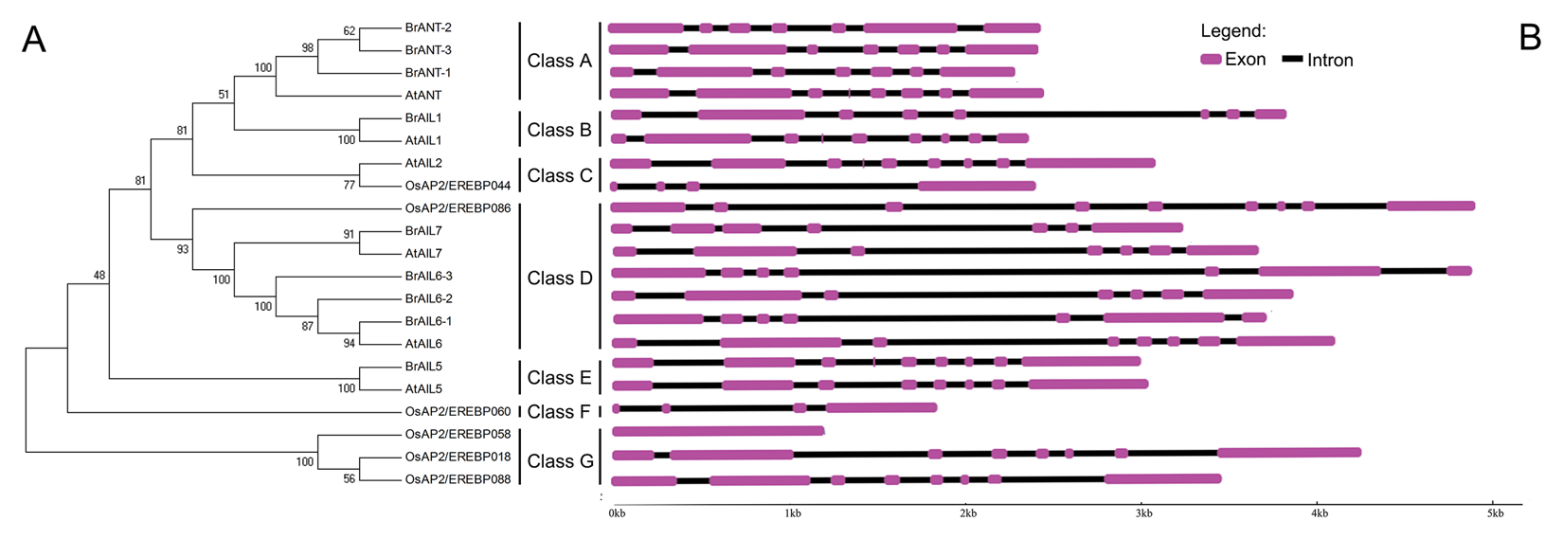
Phylogenetic relationships and intron/exon structure analysis among three species (Chinese cabbage, Rice and *Arabidopsis*). (**A**) The phylogenetic tree was constructed by MEGA5 using the Neighbor-Joining method with 1000 bootstrap replicates of the AIL proteins among Chinese cabbage, rice and *Arabidopsis*, (**B**) Intron and exon structures of the AIL family members in these three species.

In order to understand the classification of the *BrAIL* genes in Chinese cabbage, the *AIL* genes from two other model plants were selected for comparative analyses, including a model C3 monocotyledon plant (rice) and a model eudicots plant (*Arabidopsis*). A phylogenetic tree was constructed based on the full-length protein sequences of Chinese cabbage, rice and *Arabidopsis*, by using the bootstrap–neighbor-joining method. The *AIL* genes of the other two species were obtained from previous reports on *Arabidopsis*^22^ and rice^23^. According to the phylogenetic analysis, the 21 members were divided into seven groups (Class A-Class G) (Fig. 1A). Low bootstrap values were obtained because the difference of the AP2 domain sequences among these three species was small, indicating that BrAILs have high similarity to AtAILs. On the contrary, BrAIL proteins were only remotely related to the OsAIL proteins. To further explore the evolutionary relationships of the coding sequences, the structural analyses of intron/exon of the three species were performed by an online tool GSDS (http://gsds.cbi.pku.edu.cn/). It was found that most of the AIL family members in the three species had at least three introns, except OsAP2/EREBP#058, while the number of introns in the *BrAIL* genes ranged from six to eight (Fig. 1B). Furthermore, most of the genes (7 out of 9) had six introns, except *BrAIL1* and *BrAIL5*, which contained seven and eight introns, respectively.

### Syntenic analysis of the AIL family members in Chinese cabbage

To understand the evolution mechanism of the *BrAIL* genes in Chinese cabbage, the syntenic genes were analyzed between *A*. *thaliana* and *B*. *rapa* with the BRAD program (http://brassicadb.org/brad/searchSynteny.php)^24^. The results showed that nine *BrAIL* genes were from five blocks of four translocation Proto-Calepineae Karyotype (tPCK) chromosomes of the ancestor, respectively. All genes were uniformly distributed on three subgenomes, namely, less fractionized (LF), more fractionized 1 (MF1), and more fractionized 2 (MF2). Additionally, two sets of triplicated genes were found in the Chinese cabbage genomes: *BrANT-1*, *BrANT-2* and *BrANT-3* in the U block; *BrAIL6-1*, *BrAIL6-2* and *BrAIL6-3* in the R block (Table 2). The other genes (*BrAIL1*, *BrAIL5* and *BrAIL7*) were singletons. Interestingly, the tandemly duplicated gene was not found in the BrAIL family.

**Table 2:** Syntenic AILgene family members between *Arabidopsis* and Chinese cabbage.

tPCKChr	Block	Ath	Bra		
			LF	MF1	MF2
tPCK4	U	AT4G37750	Bra011782	Bra017852	Bra010610
tPCK6	E	AT1G72570		Bra008040	
tPCK5	Wb	AT5G57390			Bra020444
tPCK7	X	AT5G65510	Bra024394		
tPCK5	R	AT5G10510	Bra009026	Bra006065	Bra028584

### Multiple sequence alignment and motif analysis

The nine BrAIL proteins were used for the multiple sequence alignment. From previous studies, the euANT proteins in *Arabidopsis thaliana* or *Oryza sativa* are divided into five regions, including the pro-domain region, the R1 domain, the linker region, the R2 domain, and the post-domain region. Accordingly, all BrANT proteins were divided into five regions (Supplementary Fig. S1). Interestingly, all BrAIL proteins belonged to the euANT subgroup according to the conserved motifs, including a 10-aa insertion in the R1 domain (the euANT1 motif: NS[C/F][K/R][K/R]EG[Q/H][S/A]R) and three insertions in the pre-domain region (the euANT2, 3, and 4 motifs: [W/L]L[G/T]FSLS, PK[L/M]E[D/N]F[L/F]G, and [T/S]FGQR). Subsequent amino acid sequence analysis was carried out on the two AP2 domains of the nine AIL family members. The E-value of the forward AP2 domain (R1) matched to the full sequences varied from 2.56e-26 to 1.54e-11, and the E-value of the subsequent AP2 domain (R2) varied from 2.35e-34 to 3.85e-30. The lengths of these two AP2 domains (AP2-R1 and AP2-R2) were constant. The length of most AP2-R1 domains (70 aa) was longer than that of AP2-R2 (65 aa), except BrANT-3. The detailed description of the two AP2 domains is shown in Supplementary Table S4. Additionally, the length of the linker region was conservative (30 aa). The motif of the BrAIL members was analyzed using an online tool MEME (meme.nbcr.net/meme/intro.html), and ten motifs were identified, including motif 1, 2, 3, 4 and 10 in nine BrAIL proteins, motif 5 and 6 in eight BrAIL proteins, motif 8 in seven BrAIL proteins, and motif 7 and 9 in three BrAIL proteins, respectively (Supplementary Fig. S2).

### Expression patterns of the BrAIL family members in various organs

**Figure 2:**
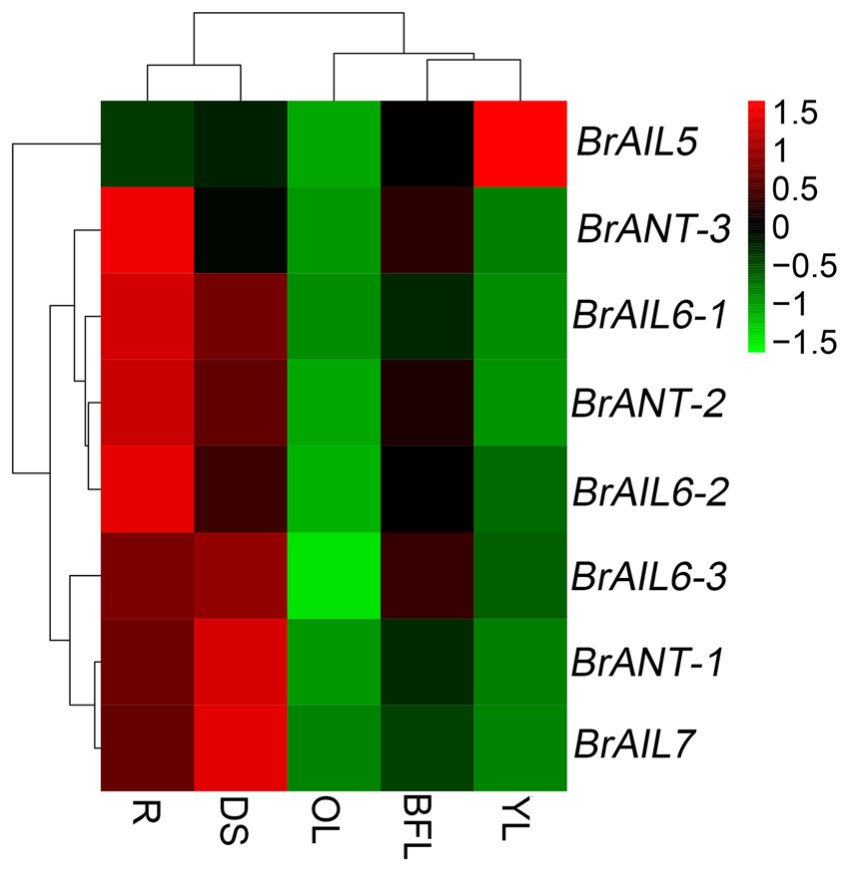
Cluster analysis of the expression pattern of the AIL family members in different tissues of Chinese cabbage. Five different tissues (50 days after sowing) were surveyed by real-time PCR, including root (R), booming flower (BFL), dwarf stem (DS), young leaf (YL) and old leaf (OL). The expression level of different tissues was calculated by the 2^−∆Ct^ method using the *BrActin* gene as the reference gene.

Accumulating experiments have shown that the *AIL* genes were expressed in multiple tissues and involved in regulating the development process of different tissues, such as root^25^, shoot^7^, floral organ^26–28^, leaf ^29^ and seed^18^. To explore the potential roles of the *BrAIL* genes in regulating the growth and development of Chinese cabbage, the expression patterns of the *BrAIL* genes was investigated in root (R), dwarf stem (DS), old leaf (OL), young leaf (YL), blooming flower (BFL) of Chinese cabbage by qRT-PCR. Eight genes were detected in different tissues, except *BrAIL1*, which was undetectable in all tissues (Fig. 2). For example, four BrAIL family members, including *BrANT-2*, *BrANT-3*, *BrAIL6-1* and *BrAIL6-2*, showed higher expression levels in R than other tissues; three *BrAIL* genes (*BrANT-1*, *BrAIL6-3* and *BrAIL7*) were mainly expressed in DS; *BrAIL5* was mainly expressed in YL. Moreover, none of the genes were detected in old leaves.

### Expression profiles of the BrAIL family members under NAA treatments

**Figure 3:**
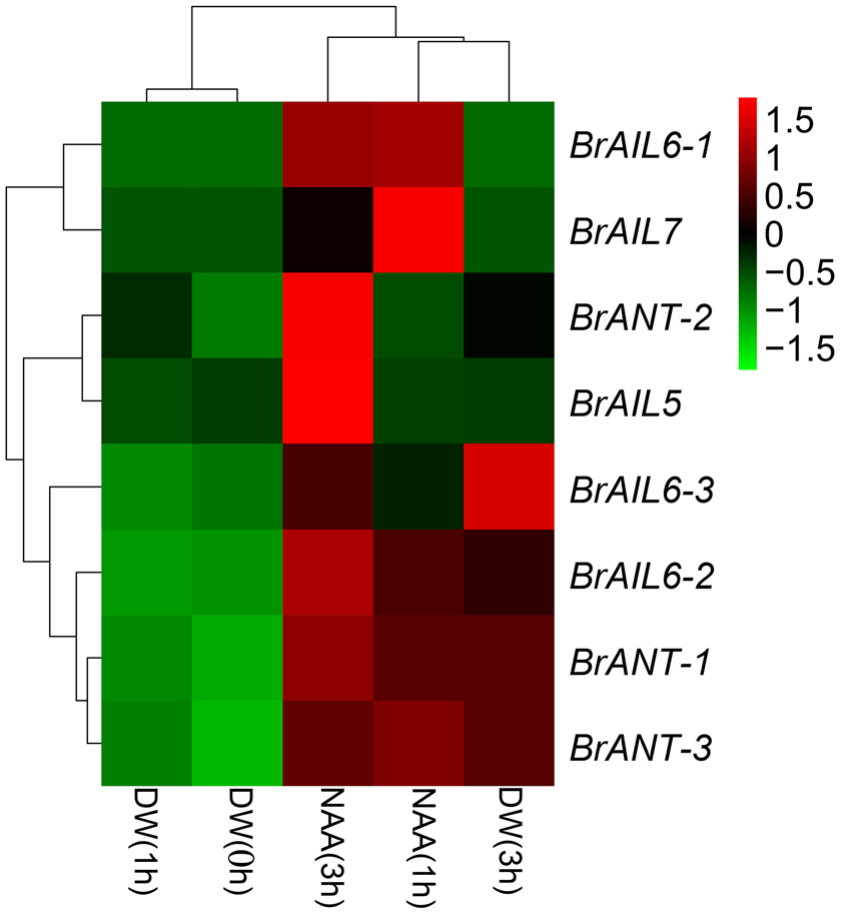
Expression profile of the BrAIL family members under naphthaleneacetic acid (NAA) treatments. Plants at the four-leafed stage (21 day after sowing) were treated with 100 μM naphthaleneacetic acid or distilled water (DW) for 0, 1, and 3 h before the leaves at the same position were harvested. Relative expression level was calculated using the 2^−ΔCt^ method. *BrActin* was used as the reference gene. The expression relative to CK was compared at 0 h.

Previous studies have shown that auxin plays an important role in plant growth and developmental processes^30^. In addition, the auxin signal may regulate organ growth by modulating *ANT* expression^10^. To understand the expression responses of the BrAIL members to auxin, we assessed the transcript levels of the *BrAIL* genes upon NAA treatments by qRT-PCR. As shown in Fig. 3, the transcriptional levels of most genes were induced by the NAA treatment, except *BrAIL1*. The expression level of *BrAIL5* was only up-regulated at 3 h. Additionally, six genes were increased at 1 h and 3 h compared with the untreated control, including *BrANT-1*, *BrANT-3*, *BrAIL6-1*, *BrAIL6-2*, *BrAIL6-3* and *BrAIL7*. However, the expression level of *BrANT-2* showed a trend of decrease at 1 h, followed by an increase at 3 h.

### Over-expression of the *BrANT-1* gene caused a variety of phenotypic changes in *Arabidopsis*

**Figure 4:**
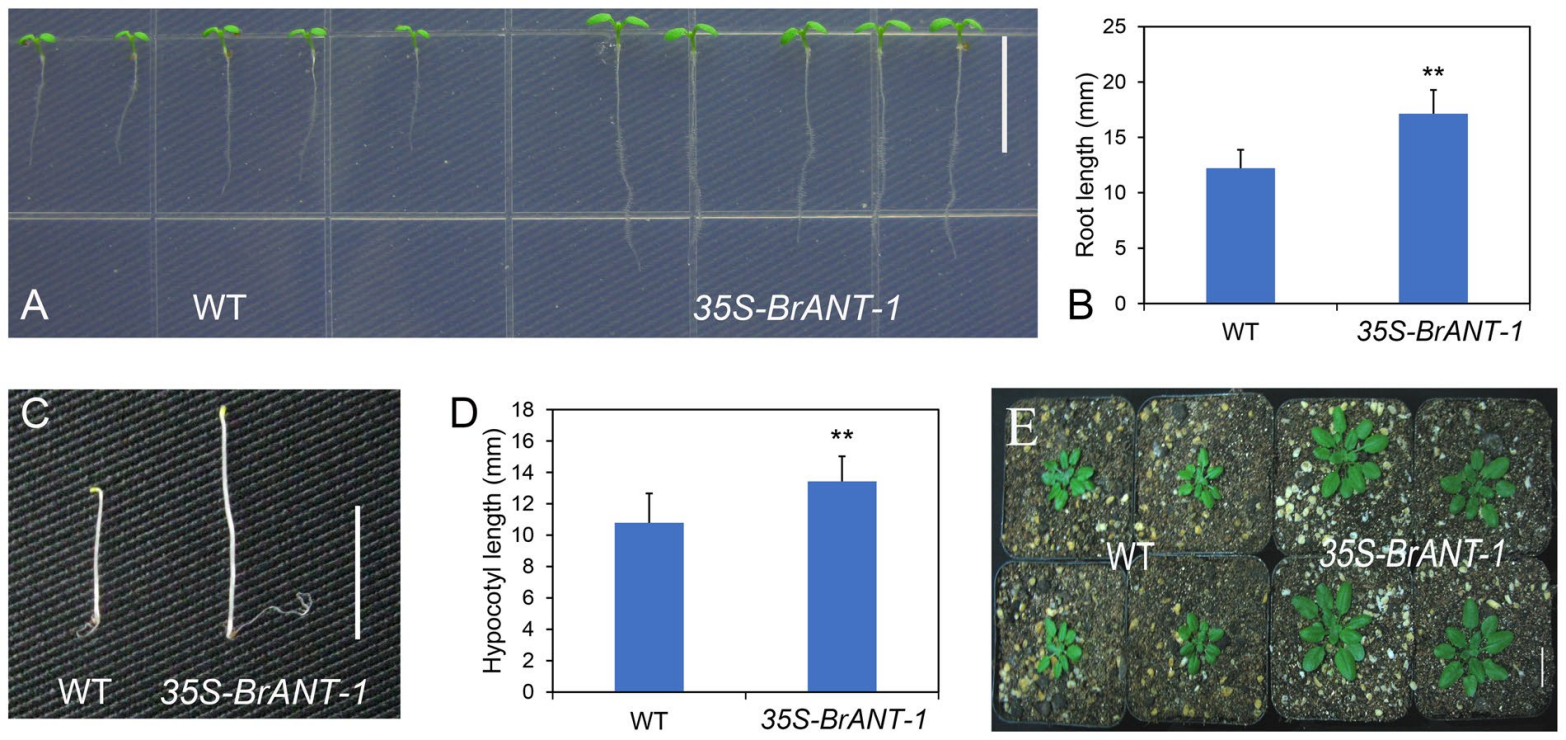
Phenotype analyses induced by overexpression of *BrANT-1* in *Arabidopsis*. (**A**) Root length of the WT and *35S-BrANT-1* transgenic plants at 7 day after sowing. Bar = 1 cm. (**B**,**D**) Root length and hypocotyl length. (**C**) Hypocotyl length of the WT and *35S-BrANT-1* transgenic plants at 3 day after germination under darkness condition. Bar = 1 cm. (**E**) The sizes of individual seedlings of the WT and *35S-BrANT-1* transgenic plants at 15 day after sowing in the nutrient soil. Bar = 1 cm. Each sample was repeated 20 times, and the error bars represent standard deviations.

**Figure 5:**
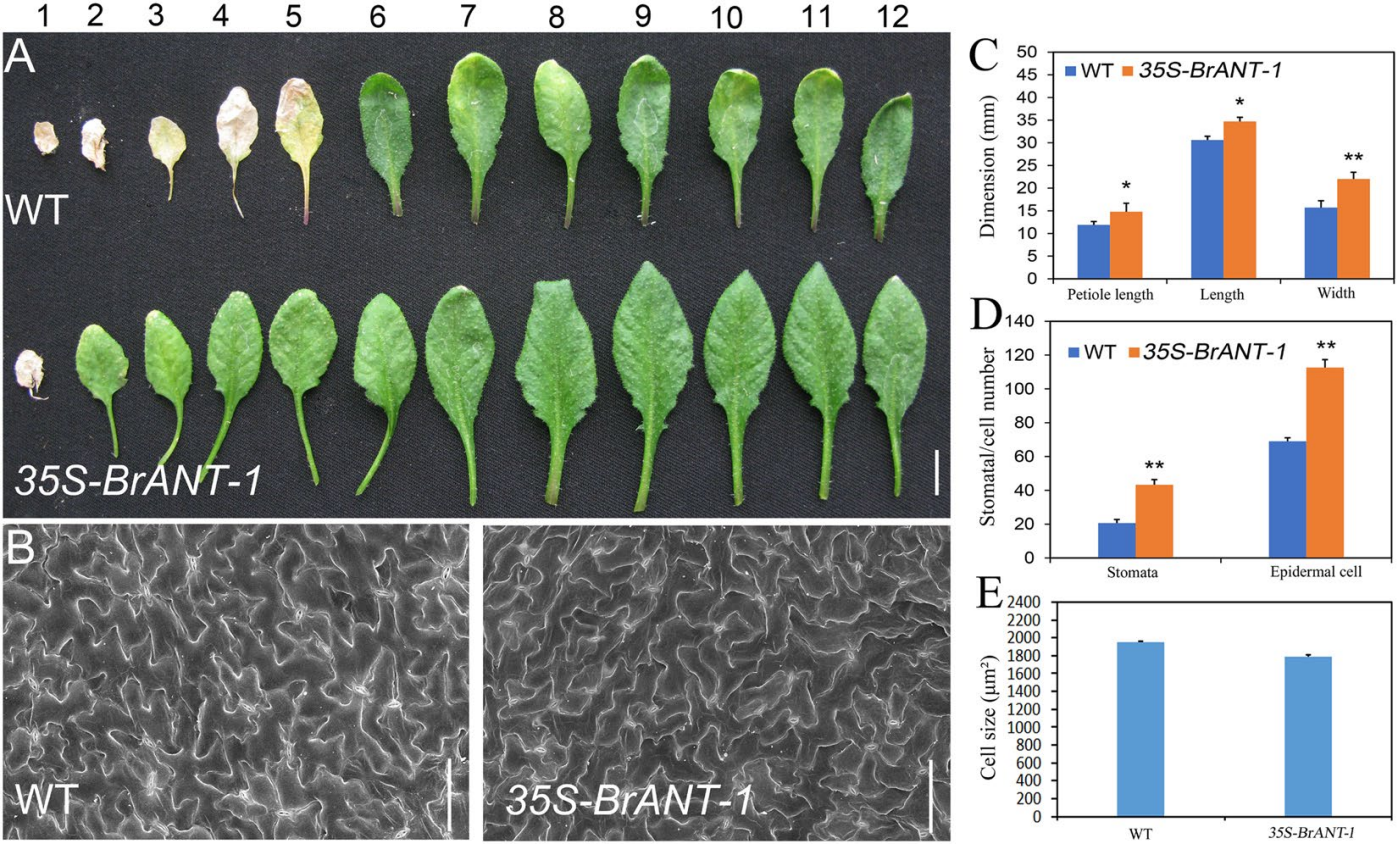
Morphology and histological analysis of mature leaves of 40 day after sowing. (**A**) The 12 leaves of the WT and *35S-BrANT-1* transgenic *Arabidopsis* at 40 day after sowing. Bar = 1 cm. (**B**) Scanning electron micrographs of the largest rosette leaves of the WT and transgenic *35S-BrANT-1* transgenic *Arabidopsis*. Bar = 100 μm. (**C**) The petiole length, leaf length and leaf width of the largest rosette leaves of the WT and transgenic *35S-BrANT-1* transgenic *Arabidopsis*. Each sample was repeated 20 times. (**D**) The number of stomata and epidermal cell per unit area (mm^2^) in the adaxial surface of fully expanded largest leaves of the WT and *35S-BrANT-1* lines. (**E**) The cell size (μm²) of the epidermal cells in the adaxial surface of fully expanded largest leaves of the WT and *35S-BrANT-1* lines. Each sample was repeated six times, and the error bars represent standard deviations. *Indicates that the expression level is significantly different from that of the control (^*^p < 0.05, ^**^p < 0.01).

**Figure 6:**
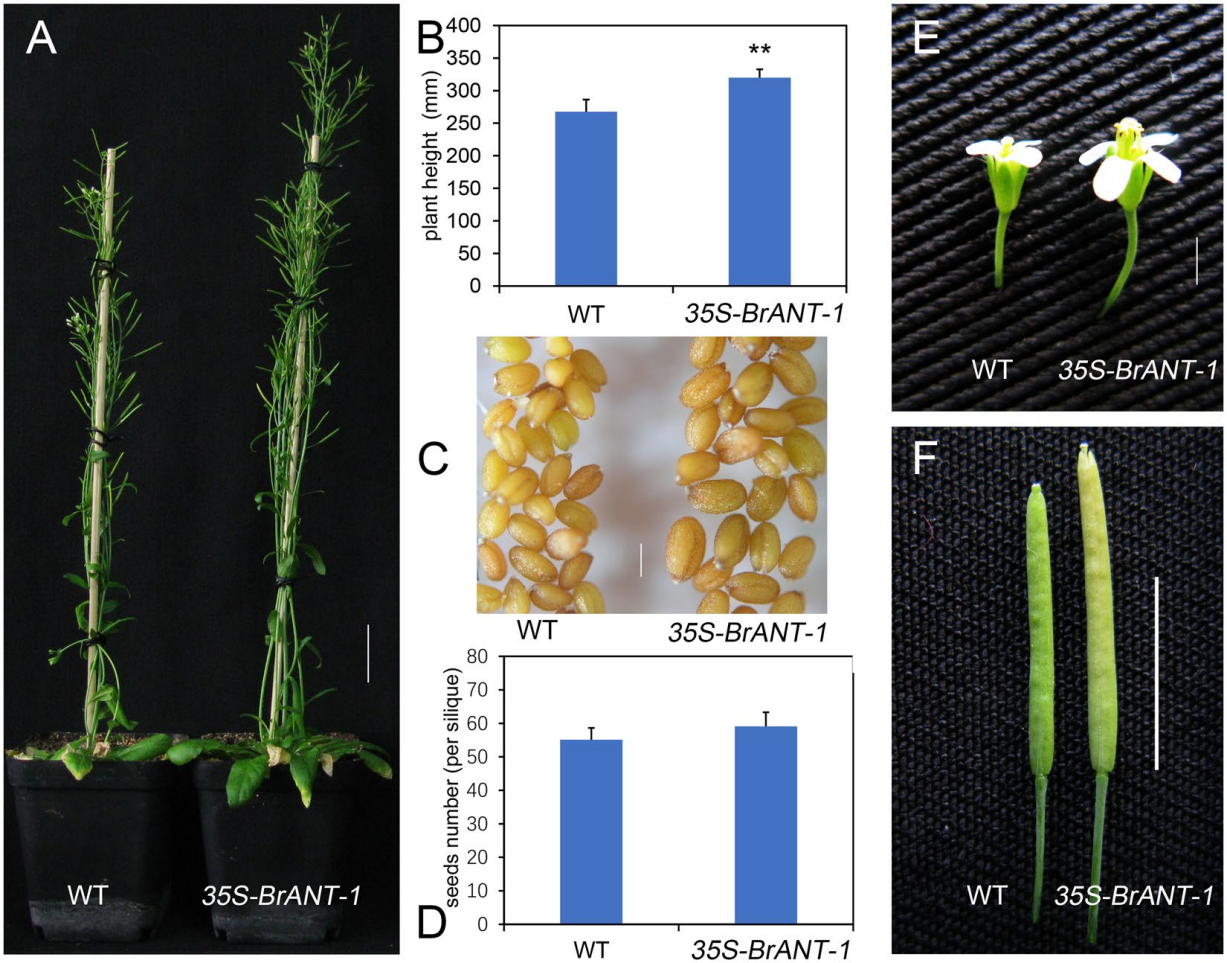
Phenotype analyses of the *35S-BrANT-1* plants induced by the overexpression of *BrANT-1* in *Arabidopsis* (40 day after sowing). (**A**) The height of the WT and *35S-BrANT-1* transgenic *Arabidopsis*. bar = 1 cm. (**B**) Plant height. Each sample was repeated 20 times. (**C**,**E**,**F**) A *35S-BrANT-1* transgenic plant produces large seed, flower and silique (**C**, bar = 100 μm; **E**, bar = 1 mm; **F**, bar = 1 cm). (**D**) Seed number (per silique). Each sample was repeated 20 times, and the error bars represent standard deviations. *Indicates that the expression level is significantly different from that of the control (^*^p < 0.05, ^**^p < 0.01); the difference in seed number per silique is not statistically significant.

Compared with other BrAIL family members, *BrANT-1* had the highest sequence similarity to *Arabidopsis thaliana ANT* (AT4G37750), while *BrANT-1* was different from other *AtAIL* genes (Supplementary Table S5). Moreover, *BrANT-1* was expressed in different tissues, suggesting that *BrANT-1* might play an important role in growth and development of Chinese cabbage. To explore the potential function of *BrANT-1*, *Arabidopsis* plants were transformed with *BrANT-1* under the control of the CaMV 35 S promoter. A series of pleiotropic effects were significantly distinguishable from the wild-type (WT) at 7 days after sowing, with the *35S-BrANT-1* transgenic seedling exhibiting a longer root (Fig. 4A). Additionally, the hypocotyl length of the transgenic seedling also showed a >24% increase compared with that of the WT (Fig. 4C). At 15 days after acclimating to the nutrient soil conditions, the seedling size of the transgenic plants was bigger than that of the WT (Fig. 4E). At 40 days after sowing, the leaf dimension of the transgenic plants was significantly increased compared with that of the WT (Fig. 5). Rachis length, flower dimension, seed size and silique were also enlarged (Fig. 6), while the seed number per silique of the *35S-BrANT-1* transgenic line was slightly more than that in the WT (Fig. 6D). Furthermore, SEM was used to compare the epidermal cells in the adaxial of mature leaves of *35S-BrANT-1* and WT. The results showed the numbers of the adaxial epidermal cells of the *35S-BrANT-1* transgenic line had a >50% increase while the cell size of the *35S-BrANT-1* transgenic line was modestly decreased (<6%) compared with the WT (Fig. 5E). However, the slightly smaller cell size could not account for the big leaf size. Therefore, we conclude that the enlarged leaf area was caused by the increase of the cell number (Fig. 5B). All phenotypic analysis data were shown in Supplementary Table S6.

Additionally, at 40 days after sowing, the transgenic lines had a delayed leaf senescence phenotype, compared with the WT, in that the fifth rosette leaf (numbered from the bottom) became senescent, while the second rosette leaf was still green (Fig. 5A).

**Figure 7:**
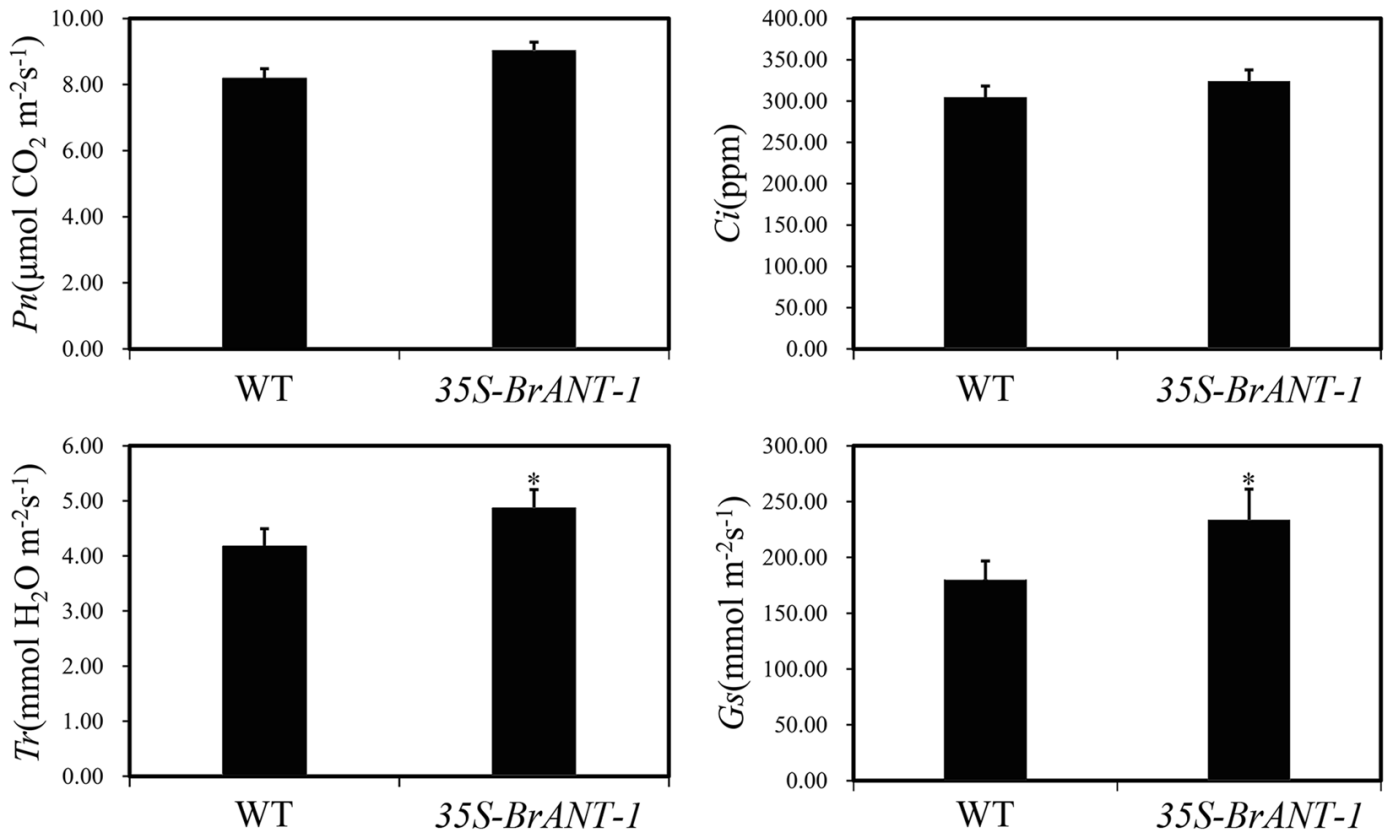
The stomatal number has a positive effect on *A*. *thaliana* leaf stomatal conductance (Gs) and transpiration rate (Tr) under the light-saturated condition. (**A**) Net photosynthetic rate (Pn) and stomatal conductance (Gs); (**B**) Intracellular CO_2_ concentration (Ci); (**C**) Transpiration rate (Tr); (**D**) Stomatal conductance (Gs). Photosynthetic capacity analysis of mature leaves of the WT and *35S-BrANT-1* transgenic plants (40 day after germination) measured using a GFS-3000 gas exchange system under the light-saturated condition (PPFD = 500 μmol m^−2^ s^−1^), and CO_2_ concentration was set to 400 ppm. Each sample was repeated 20 times, and the error bars represent standard deviations. *Indicates that the expression level is significantly different from that of the control (^*^p < 0.05).

In addition, other interesting phenotypes were observed. For example, the stomatal density of the *35S-BrANT-1* line was significantly increased in the mature leaves compared with that of the WT (Fig. 5B, detailed in Supplementary Fig. S3). Consistently, the stomatal conductance (Gs) and transpiration rate (Tr) of the 40-day *35S-BrANT-1* plants were significantly increased compared with those of the WT, while the intracellular CO_2_ concentration (Ci) and net photosynthetic rate (Pn) between the WT and *35S-BrANT-1* plants were only slightly changed (Fig. 7).

### Transcriptome sequencing of the *35S-BrANT-1* and WT plants

**Figure 8:**
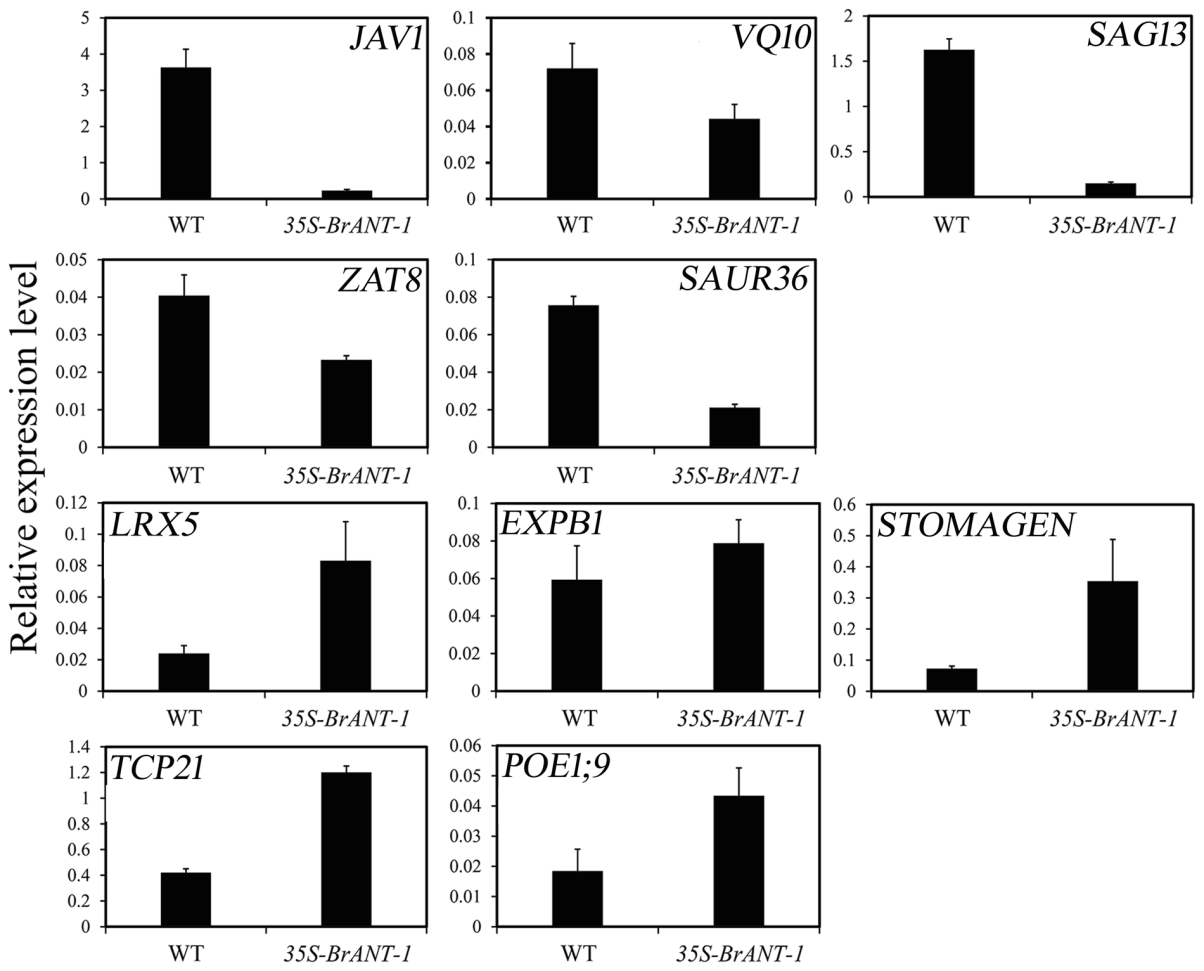
qRT-PCR validation of the gene expression from RNA-Seq analyses. Ten DEGs (*JAV1*, AT3G22160; *SAG13*, AT2G29350; *VQ10*, AT1G78410; *ZAT8*, AT3G46080; *SAUR36*, AT2G45210; *LRX5*, AT4G18670; *EXPB1*, AT2G20750; *STOMAGEN*, AT4G12970; *TCP21*, AT5G08330; *POE1;9*, AT5G15780) were randomly selected for further verification by qRT-PCR. Relative expression levels were calculated using *AtActin* (At2g37620) as the reference gene by the method 2^−ΔCt^. Data presented are mean values of three biological replicates, and the error bars represent standard deviations.

To investigate the genome-wide effects of *BrANT-1* on transcription, transcriptome sequencing was carried out for the WT and *35S-BrANT-1* in three independent biological replicates. A total of 533 differentially expressed genes (DEGs) were identified between the WT and *35S-BrANT-1* plants, including 94 up-regulated genes and 439 down-regulated genes (Supplementary Table S7). Additionally, ten DEGs were randomly selected for further verification by qRT-PCR; all genes exhibited the same expression tendency as shown in the original data (Fig. 8). According to the *Arabidopsis* genome sequence and the DEGs could be assigned to different families, such as MADS-box, *TCP*, *extension*, *expansin*, early auxin-responsive genes (small auxin-up RNA, *SAUR*), *VQ*, *STOMAGEN*, *SAGs* and transcription factors. The DEGs that were related to plant growth and development were selected and characterized (Table 3). Further analysis indicated many genes were up-regulated in 35S-BrANT-1, which were mainly involved in cell proliferation (MADS-box protein, AT1G59920; *TCP21*, AT5G08330) and stoma development (*STOMAGEN*, AT4G12970). On the other hand, the genes inhibiting plant growth (*SAUR33*, AT3G61900; *VQ22*, AT3G22160; VQ3, AT1G21326 and *ANAC036*, AT2G17040) and promoting leaf senescence (*AtNAC2*, AT5G39610; *SUAR36*, AT2G45210; *SAG13*, AT2G29350) were down-regulated in the *35S-BrANT-1* line.

**Table 3:** List of up/down-regulated genes related to pleiotropic phenotypes (Cell growth /Cell wall, Plant growth and development, and Stoma development and Senescence related gene) in the *35S-BrANT-1* transgenic *Arabidopsis* line.

Gene ID	Short description	Functions	localization	Transcript level	Regulated
Cell growth/Cell wall					
AT1G59920	MADS-box family protein	Unknown	nucleus	9.311	Up
AT5G20630	GLP3	Primary cell walls	Cell wall	5.908	Up
AT5G15780	POE1;9	Unknown	Endomembrane system	3.736	Up
AT2G20750	EXPANSIN B1	Cell wall loosening	Cell wall	3.476	Up
AT4G18670	LRX5	Structural constituent of cell wall	Endomembrane system	3.023	Up
AT5G13140	POE1;23	Unknown	Mitochondrion	2.892	Up
AT3G16670	POE1;5	Unknown	Extracellular region	2.596	Up
AT2G45470	FLA8	Cell wall composition	Plant-type cell wall	2.063	Up
AT2G06850	XTH4	Cell wall structure and composition	Plant-type cell wall	1.424	Up
AT5G08330	TCP21	Unknown	nucleus	1.416	Up
Plant growth and development					
AT1G56150	SAUR71	Unknown	cytoplasm	−2.683	Down
AT3G12830	SAUR72	Unknown	cytoplasm	−2.597	Down
AT3G61900	SAUR33	Antinutritiver factor	nucleus	−2.269	Down
AT3G15300	MVQ4	Antinutritiver factor	nucleus	−2.963	Down
AT3G22160	JAV1/VQ22	Antinutritiver factor	nucleus	−1.907	Down
AT1G21326	VQ3	Antinutritiver factor	nucleus	−1.79	Down
AT3G60690	SAUR59	Unknown	mitochondrion	−1.776	Down
AT2G17040	ANAC036	Antinutritiver factor	nucleus	−1.438	Down
Stoma development					
AT4G12970	STOMAGEN/ EPFL9	Postive regulator of stoma density	Epidermis cell	1.964	Up
Senescence related gene					
AT2G29350	SAG13	Short-chain alcohol dehydrogenase	chloroplast	−2.452	Down
AT5G39610	AtNAC2	Positively regulates aging-induced cell death and senescence in leaves	nucleus	−2.264	Down
AT2G45210	SAUR36/SAG201	Inhibit growth/ Positive regulation of leaf senescence	mitochondrion, nucleus	−3.182	Down

Transcript level, transcript level ratio between *35S-BrANT-1* lines and WT.

## Discussion

### *AIL* genes duplication in Chinese cabbage

The genome of the mesopolyploid crop species *Brassica rapa* has undergone whole genome triplication (WGT) since divergence from *Arabidopsis thaliana*, resulting in a significant increase of the numbers of the duplicated genes^31^. However, only nine AIL family members were identified in Chinese cabbage, including three *BrANTs* and six *BrAILs*, which were only 1.5-fold more than those in *Arabidopsis*, suggesting many genes were lost during genome duplication^32^. Similar results were also found in other gene families, such as *BrGRF* and *BrVQ* genes, which were about 1.89- and 1.9-fold more than those in *Arabidopsis*, respectively^13,33^. Additionally, the expansion of the BrAIL family members mainly depended on the segmental duplication, because no tandem duplicated pairs were found. Many studies on genome duplications have shown that the genes involved in transcription, protein binding, response to biotic stimuli and signal transduction path are preferentially retained by segmental duplication^34–36^. Duplication events within a genome can result in paralogs, and these genes may have different expression patterns following duplication indicative of subfunctionalization^37^. For example, two duplicated genes, *BrVQ22-1* and *BrVQ22-2*, are differentially expressed in different tissues^33^. In this study, the triplicated genes of *BrAIL6-1/-2/-3* and *BrANT-1/2/3* also exhibited a different expression pattern in different organs and in response to the auxin treatment. Additionally, similar cases have also been reported in the AP2/ERF family in Chinese cabbage^38,39^.

### BrAIL family members were expressed in various tissues

By quantitative real time PCR, we have shown that most of the BrAIL family members were differentially expressed in both vegetative and reproductive tissues (Fig. 2). Similar to the *AtAIL* genes, the *BrAIL* genes had higher expression in young tissues (seedling and roots) and lower expression or absent in mature leaves^40^. Multiple *BrAIL* genes were expressed in different tissues, suggesting that the *BrAIL* genes play different roles in different organs, which has been confirmed in other plants. For example, *ANT* and *AIL6* are involved in the regulation of flower or seed development in Arabidopsis or Medicago truncatula^9,17,18^ and the *VviANT1* gene plays an important role in the regulation of berry size^28^. Additionally, we observed the *BrAIL* genes were lowly expressed in blooming flowers. A previous study also found that the *AIL* genes are mainly expressed in the advanced stage B (B2), flowers from inflorescences at stage G (G), and the early stage H (H1) in grapevine, while the expression of the *AIL* genes is low in blooming flowers^28^. Besides, *AtANT*, *AtAIL5*, *AtAIL6*, and *AtAIL7* also exhibit the same expression tendency during the flower development^40^.

### Transgenic lines over-expressing *BrANT-1* had enlarged organs by increasing the cell number

Generally, the *AIL* genes play an important role in regulating organ growth through increasing cell number^7^ or cell size^29^. In the present study, the cell number in the leaf was increased in the *35S-BrANT-1* transgenic lines, implying that *BrANT-1* positively regulates cell proliferation. This result was consistent with the function of the *AtANT* gene in the leaf or floral organ^7^. However, it is different from the roles of *PnANTL1* and *PnANTL2* genes, which increase leaf length through increasing cell size in tobacco^29^.

Recently, Liu *et al*.^41^ found that the *OsMADS1* gene can positively regulate cell proliferation in rice. Therefore, the most up-regulated *MADS-box* gene (AT1G59920) among the DEGs may play critical roles in increasing the cell number in the *35S-BrANT-1* line. Additionally, previous studies have shown that the *TCP* gene can be divided into two classes (I and II). The class I genes like *TCP20* function as positive regulators of cell growth^42^, while the class II genes like the Antirrhinum genes *CINCINNATA* (*CIN*) function as negative regulators of cell growth^43^. Here, *AtTCP21* (AT5G08330), a member of the class I genes^44^ was found to be up-regulated, suggesting that it may partially promote cell proliferation in the *35S-BrANT-1* line. Apart from the up-regulated genes, some down-regulated genes, such as *SAUR36* (AT2G45210)^45^, *VQ22* (AT3G22160)^46^ and *ANAC036* (AT2G17040)^47^, might also play an important role in increasing organ size in the *35S-BrANT-1* line, as indicated by previous studies, which have shown that some *SAUR*, *VQ* and *NAC* genes are involved in the negative control of plant growth^46–50^. Interestingly, some genes, such as *LRX5* (AT4G18670)^51^ and *expansinB1* (AT2G20750)^52^, positively controlling cell size were also identified from the up-regulated DEGs, as indicated in our histological results which showed a slight reduce in cell size between the *35S-BrANT-1* and WT plants. The result was consistent with our previous studies on the *BrARGOS* gene, which regulates the *ANT* gene^10^ and may also promote the transcription of *AtEXP10* in transgenic Arabidopsis^53^. This is probably because meristematic competence is disrupted locally, cell division gradually ceases and differentiation begins with the expansion of the postmitotic cells^19^. In addition, cell proliferation is also coupled with a limited amount of cell expansion during the proliferation phase^54^. Therefore, we speculate that the extensin and expansin proteins promote cell expansion following a significant increase of cell proliferation. The modest reduction in cell size may compensate the increase in cell number^19^.

### *BrANT-1* might regulate leaf senescence

Leaf senescence constitutes the final phase of leaf development and is a highly complex but genetically programmed process involving the expression of many senescence-associated genes (*SAGs*)^55,56^. Our study shows that, compared with the wild type, leaf senescence in the *35S-BrANT-1* transgenic line was delayed, which is consistent with that in *Arabidopsis* over-expressing the *AtANT* gene^57^. Additionally, a number of SAGs such as *AtNAC2* (AT5G39610)^58^, *AtSAUR36*(AT2G45210)^45^, and *AtSAG13*(AT2G29350)^59^ were prominently down-regulated in the *35S-BrANT-1* line. *AtSAUR36* (or *SAG201*), a member of the early auxin-responsive gene family, was remarkably up-regulated during leaf senescence. In *Arabidopsis*, a saur36 knockout line shows a delayed leaf senescence phenotype, but the transgenic plant overexpressing *SAUR36* displays an opposite phenotype^45^. In addition, *SAG13* as an early senescence marker is strongly induced before visible yellowing^60^. Accordingly, we speculate that *BrANT-1*, similar to the *AtANT* gene, is one of the negative factors that prevent premature senescence.

### *BrANT-1* positively increased the stomatal number in *Arabidopsis*

Stomata is an important part of the epidermal tissues of leaves in plants, which controls gas exchange by paired subsidiary cells, and participates in the global carbon cycle^61^. Stomatal development in leaf is positively regulated by signaling factor *STOMAGEN* (At4g12970) through interacting with cell-surface receptor *TOO MANY MOUTHS* (TMM)^62^. In this study, it was found that the number of stoma significantly increased in the *35S-BrANT-1* line compared with the WT. Besides, the expression level of *STOMAGEN* (At4g12970) was significantly up-regulated in the transgenic line. The overexpression of *STOMAGEN* was found on many agminate stomata in mature leaves of *Arabidopsis*^62^. However, the homologous gene of *AtANT* regulating the stomatal development has not been reported. Additionally, previous studies have shown that the stomatal density in *Arabidopsis* influences the leaf photosynthetic capacity through regulating gas diffusion^63^. In this study, we assessed the leaf photosynthetic capacity of the *BrANT-1*-overexpressing transgenic and wild type lines. The result indicated that, with the increase of the stomatal number, the stomatal conductance (Gs) and transpiration rate (Tr) of mature leaves were significantly increased in the *35S-BrANT-1* transgenic plants. However, the net photosynthetic rate (Pn) was only slightly increased in the *35S-BrANT-1* plants, probably due to the low intracellular CO_2_ concentration (Ci). Severe stomatal patchiness results in the underestimation of Pn due to the lack of uniform Ci in the leaf ^64^. Taken together, these results indicated that *BrANT-1* might be involved in regulation of the development of stomata by enhancing the expression level of *STOMAGEN* (At4g12970).

In summary, we identified three BrANT and six BrAIL proteins in Chinese cabbage, which can be classified into four subgroups. Phylogenetic analysis showed that the *AIL* genes of Chinese cabbage had high sequence similarity with those of Arabidopsis. Furthermore, multiple sequence alignment suggests that the nine genes belonged to the euANT subgroup according to the conserved motifs in Arabidopsis. Finally, ectopic expression of *BrANT-1* in Arabidopsis controlled the organ size by regulating the cell number. *BrANT-1* regulated the stomatal density and leaf senescence by increasing the expression of *STOMAGEN* and reducing the expression of *SAGs*. Taken together, these results not only enhance the understanding of the role of the *AIL* genes in controlling the organ size and other tissue growth, but also provide a promising tactic for Chinese cabbage molecular breeding program.

## Methods

### Identification and analysis of the *AIL* genes in Chinese cabbage

The gene and amino acid sequences of the AIL family members were confirmed according to the genome of the *B*. *rapa* line Chiifu (http://brassicadb.org). The difference of the *AIL* genes was analyzed using DNAMAN 6.0.40 (Lynnon Biosoft, USA). Gene Structure Display Server (GSDS) (http://gsds.cbi.pku.edu.cn/) was used to perform the intron/exon structure analysis. Phylogenetic trees were constructed based on the amino acid sequences using the neighbor-joining method by MEGA 5.0 software^65^. The physicochemical properties of the AIL proteins were calculated by using the ProtParam tool (http://web.expasy.org/protparam/). MEME (meme.nbcr.net/meme/intro.html) was used to analyze the conserved motifs of the AIL proteins in Chinese cabbage^66^. The AP2 domain sequences of the AIL proteins from Chinese cabbage were identified with SMART (http://smart.embl-heidelberg.de/).

### Plant materials, growth conditions and plant phytohormone treatments

The wild-type and *35S-BrANT-1* transgenic *Arabidopsis* plants were pre-treated for 3 days at 4 °C under dark condition before transferring to pots with nutrient media (Sheng Xiang Agricultural Science and Technology Co, China; peat: vermiculite: pearlite = 1: 1: 1). The plants were cultivated in a growth room with a continuous artificial light period of 16 h and a dark period of 8 h, and a constant temperature between 19–23 °C. The Chinese cabbage line “Guangdongzao” was used for all stress experiments, and the growth condition was the same as that for *Arabidopsis*.

Chinese cabbage young seedlings at the four-leafed stage (21 days after sowing) were used for the phytohormone treatments, during which, the plant leaves were treated with 100 μM naphthaleneacetic acid or distilled water (DW), respectively. Plant leaves were harvested after 0, 1 and 3 h of the phytohormone treatment. For gene expression analysis of *BrANTs* in different tissues, the plants were pre-treated for 15 days at 4 °C under dark condition to accelerate the transition from the vegetative phase to the reproductive phase. Plant organs were harvested after the plants bloomed (50 days after sowing, including the pre-treated time), including root (R), dwarf stem (DS), old leaf (OL), young leaf (YL), and blooming flower (BFL). RNA preparation was performed for each organ in three biological replicates.

### Plant transformation

The complete coding region (1671 bp) of Chinese cabbage ANT cDNA was amplified by using the following primers: BrANT-1-forward: 5′-GT*TCTAGA*ATGAAGTCCTTTTGTGATAATGATG-3′ and BrANT-1-reverse: 5′-TA*GTCGAC*TCAAGAATCAGCCCACGCAGCGAAA-3′. The italic sequences (*TCTAGA and GTCGAC*) were marked as the restriction sites for *XbalI* and *SalI*, respectively. The CAMV 35 S promoter was used instead of the intrinsic promoter. The product of polymerase chain reaction (PCR) was cloned into the binary vector pCAMBIA2300-35SOCS using the *XbalI* and *SalI* restriction sites. The constructed vector was used to transform *Arabidopsis* plants using the planta Agrobacterium-mediated method^67^ and the transgenic seedlings were selected by 1/2 MS agar plates containing 50 mg/mL kanamycin sulfate. Homozygous transgenic plants were used for further analyses.

### RNA isolation and cDNA synthesis

The total RNA of each sample (in three biological replicates) was isolated using Trizol reagent (Invitrogen, Carlsbad CA, USA). The quality and concentration of the total RNA were measured separately using a 2100 Bioanalyzer RNA Nanochip (Agilent, Santa Clara, CA, USA) and a NanoDrop ND-2000 Spectrophotometer (Nano-Drop, Wilmington, DE, USA). The synthesis of cDNA was carried out using a PrimeScript™ RT reagent kit with a gDNA Eraser (Takara, Dalian, China).

### Sequencing and data processing

The matured leaves of the WT and *35S-BrANT-1* transgenic *Arabidopsis* plants (40 days after sowing) were used for RNA-seq. Each line was biologically repeated three times. The sequencing of six cDNA libraries was performed at Beijing Genomics Institute (BGI, Shenzhen, China) using an Illumina HiSeqTM 2000 sequencing platform (Illumina Inc., San Diego, CA, USA). Clean reads were obtained from the raw reads that were clipped by abandoning the adaptor sequences and low-quality reads (reads with >10% ambiguous “N” bases or reads in which >50% of the bases had a Quality-score ≤ 5). The clean reads were mapped to the reference genome using a rapid short-read mapping program, namely SOAP aligner/soap2^68^. More than two mismatches were abandoned in the sequence alignment. The quality of sequencing was controlled by quality assessment of reads, statistics of alignment, sequencing saturation analysis and randomness assessments.

### Identification and functional annotation of differentially expressed genes

The reads per kb per Million reads (RPKM) method^69^ was used to calculate the expression level of each unigene. Therefore, the RPKM values can be directly used for comparing the difference of gene expression among the WT and *35S-BrANT-1* lines. The DEGs from these two lines (six cDNA libraries) were enriched for further analysis according to the standard with false discovery rate (FDR) ≤0.01, and the absolute value of log2 ratio ≥ 1.

### Quantitative RT-PCR (qRT-PCR) analysis

To confirm the quality of the sequencing data and distinguish the expression level, the Chinese cabbage *AIL* genes were subjected to qRT-PCR analysis. qRT-PCR was performed under the following conditions: 94 °C for 2 min, followed by 45 cycles of reaction (94 °C for 20 s, followed by 60 °C for 34 s). The actin gene was used as a constitutive expression control. qRT-PCR was performed on an IQ5 Real-Time PCR System (BIO-RAD, Hercules, CA, USA). The specific primers for qRT-PCR (Supplementary Table S8) were designed using Primer Premier 5.0 (Premier Biosoft International, Palo Alto, CA) and synthesized by Shanghai Sangon Biological Engineering Technology & Services Company (Shanghai, China.). Three replicates were performed for each sample.

### Scanning electron microscopy analysis

To observe the morphology of leaf blades, leaf sections were cut from the fully expanded eighth leaf of the WT and *35S-BrANT-1* plants (40 days after sowing), embedded in 100 mM sodium phosphate buffer (pH 7.2) containing 2% glutaraldehyde for 2 h at 4 °C. Then the samples were rinsed for 1 h by the same buffer and dehydrated in a graded ethanol series for 1 h at each gradation. The dehydrated samples were dried in a critical-point dryer with liquid CO_2_ as the transitional fluid and examined using a scanning electron microscope (SEM; JEOL JSM-7600 F, Japan) after coated with gold. The size and number of the epidermal cells on the adaxial side were determined in the middle region of a half leaf near the midvein. At least six leaves from each of the WT and transgenic plants were selected for cell number counting in a fixed area on the SEM images.

### Data availability

The RNA-seq raw data are deposited in the Sequence Read Archive (SRA) under the number “SPR136061”. Phenotype datasets are available in this article and its supplementary files.

## Electronic supplementary material

Table S1

Table S2

Table S3

Table S4

Table S5

Table S6

Table S7

Table S8

Supplementary Figure
