# Supplementary material for: Ectopic expression of a Brassica rapa AINTEGUMENTA gene (BrANT-1) increases organ size and stomatal density in Arabidopsis
Source: Sci Rep. 2018 Jul 12;8:10528. doi: 10.1038/s41598-018-28606-4 (PMC6043548; doi:10.1038/s41598-018-28606-4)
Supplement: Supplementary file 9 — Supplementary Figure [file 41598_2018_28606_MOESM9_ESM.pdf]

Supplementary Material

Ectopic expression of a *Brassica rapa AINTEGUMENTA* gene (*BrANT-1*) increases organ size and stomatal density in Arabidopsis

Qian Ding[1], Bing Cui[1,2], Jingjuan Li[1], Huayin Li[1], Yihui Zhang[1], Xiaohui Lv[1], Nianwei Qiu[1,3], Lifeng Liu[1], Fengde Wang[1], Jianwei Gao[1]

[1]Institute of Vegetables and Flowers, Shandong Academy of Agricultural Sciences and Shandong Key Laboratory of Greenhouse Vegetable Biology and Shandong Branch of National Vegetable Improvement Center, Jinan 250100, China

[2]College of Life Sciences, Shandong Normal University, Jinan 250014, China

[3]College of Life Sciences, Qufu Normal University, Qufu 273165, China.

Qian Ding and Bing Cui contributed equally to this work.
Correspondence and requests for materials should be addressed to F.-D.W. (wfengde@163.com) and J.-W.G. (jianweigao3@yahoo.com).

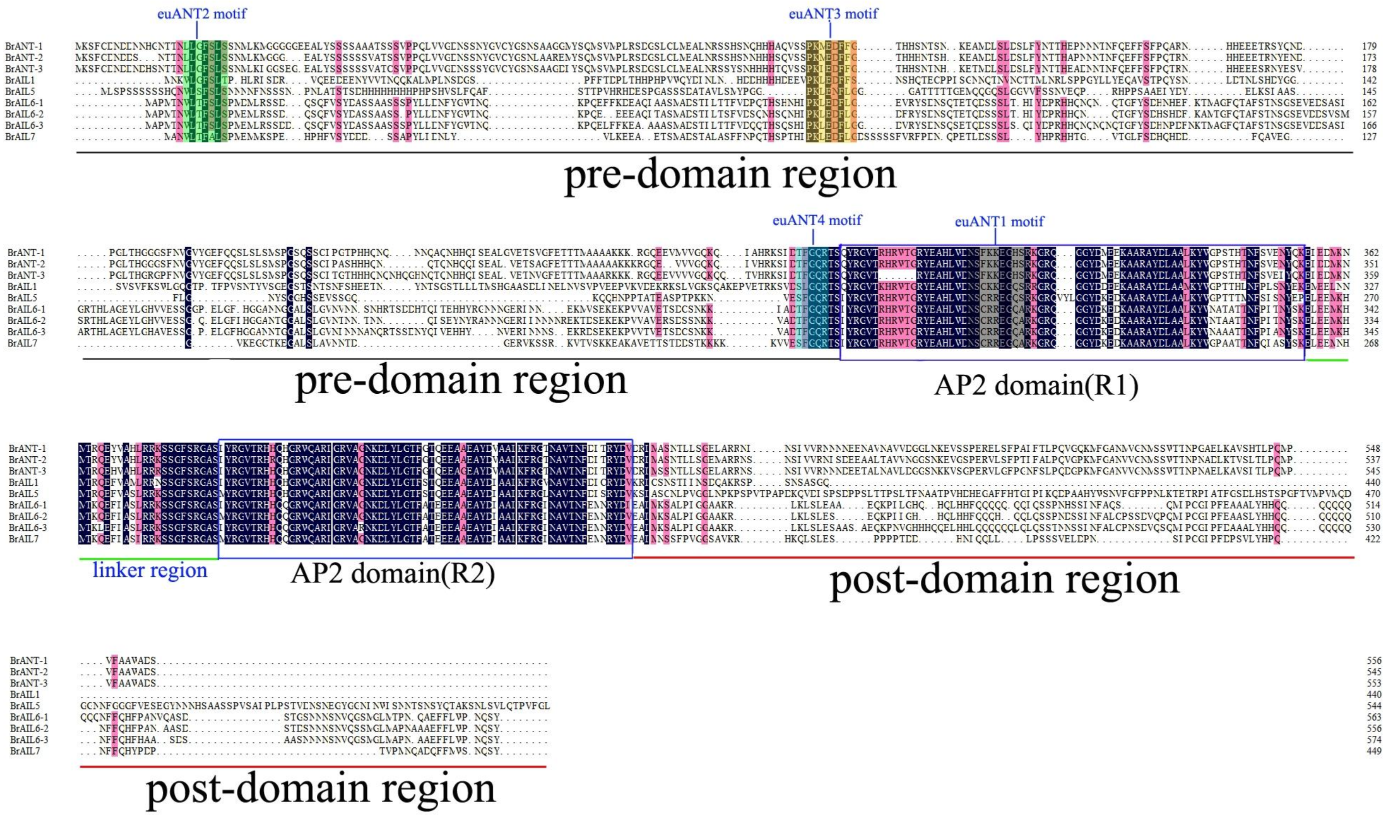

Supplementary Figure S1. Multiple sequence alignment of the BrANT lineage proteins. The alignment was performed by the DNAMAN software. All BrANT lineage protein sequences were divided into five domains, including pre-domain region, AP2 domain (R1), linker region, AP2 domain (R2) and post-domain region.

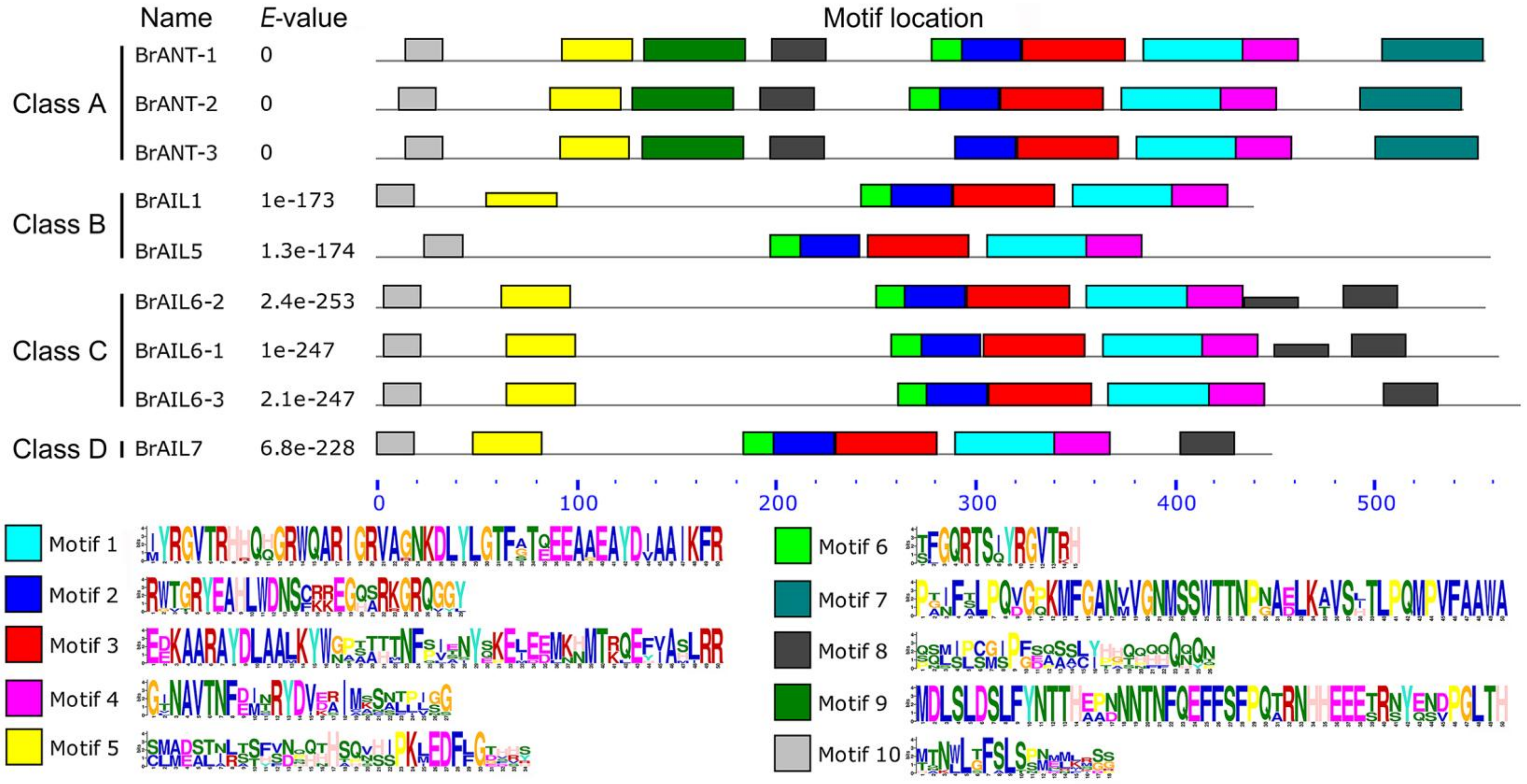

Supplementary Figure S2. Distribution of the conserved motifs in the BrANT-lineage proteins. Motif analysis was performed by the online tool MEME (meme.nbcr.net/meme/intro.html).

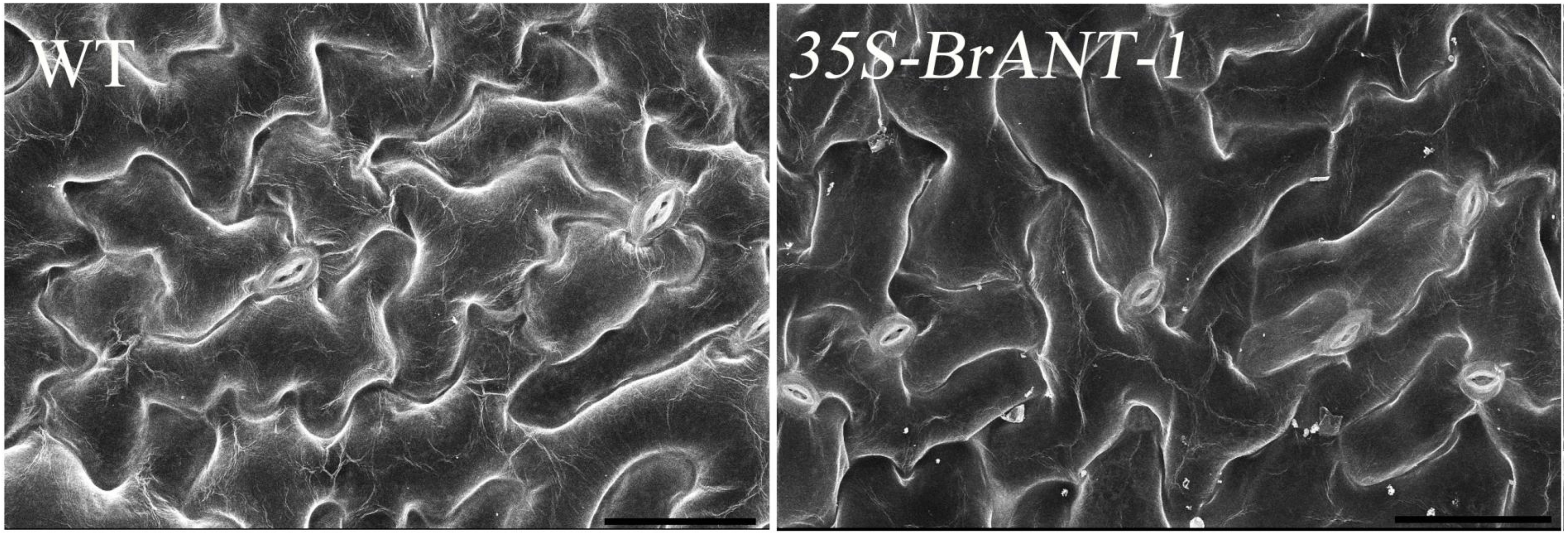

Supplementary Figure S3. Scanning electron micrographs of the largest rosette leaves of the WT and transgenic *35S-BrANT-1* transgenic *Arabidopsis*. Bar=500 μm.
